# Prevalence and associated factors of malnutrition among adults living with HIV on ART: a pilot study

**DOI:** 10.1016/j.metop.2026.100483

**Published:** 2026-06-30

**Authors:** Panagiotis Provias, Eleni C. Pardali, Aleks Pepa, Arriana Gkouvi, Dimitrios Poulimeneas, Christina Tsigalou, Maria Dalamaga, Dimitrios G. Goulis, Dimitrios P. Bogdanos, Maria G. Grammatikopoulou

**Affiliations:** a Department of Nutritional Sciences & Dietetics, Faculty of Health Sciences, International Hellenic University, Alexander Campus, Sindos, P.O. Box 141, Thessaloniki, GR-57400, Greece; b Immunonutrition and Clinical Nutrition Unit, Department of Rheumatology and Clinical Immunology, Faculty of Medicine, School of Health Sciences, University of Thessaly, Biopolis, Larissa, GR-41223, Greece; c Department of Food Science and Human Nutrition, Agricultural University of Athens, 75th Iera Odos Str., Athens, GR-11855, Greece; d Department of Nutritional Science and Dietetics, School of Health Sciences, University of the Peloponnese, Kalamata, GR-24100, Greece; e Department of Nutrition and Dietetics, Harokopio University, 70th El. Venizelou Avenue, Kallithea, Athens, GR-17671, Greece; f Laboratory of Hygiene and Environmental Protection, Medical School, Democritus University of Thrace, University Hospital, Alexandroupolis, GR-68100, Greece; g Department of Biological Chemistry, School of Medicine, National and Kapodistrian University of Athens, Athens, GR-11527, Greece; h Unit of Reproductive Endocrinology, 1st Department of Obstetrics and Gynecology, Faculty of Health Sciences, Medical School, Aristotle University of Thessaloniki, Thessaloniki, GR-54124, Greece

**Keywords:** AIDS, HIV, Nutritional status, Wasting, Appetite loss, Stavudine, Zidovudine, Isoniazid, Tenofovir alafenamide, Lipodystrophy, Malnutrition, Obesity, Dietary intake, Immunonutrition, Diet, Nutritional knowledge, Depression, Mental health, Physical activity

## Abstract

**Introduction:**

Antiretroviral therapy (ART) is widely used in the management of people living with human immunodeficiency virus (HIV) and has substantially improved clinical outcomes and reduced mortality. However, despite these benefits, individuals receiving ART remain at risk of malnutrition. This pilot study aimed to assess the prevalence of malnutrition and depressive symptoms, as well as patterns of physical activity and appetite, among people living with HIV (PWH) on ART, in a Greek population.

**Methods:**

A total of 44 PWH on ART participated in the study. Malnutrition was evaluated using three validated screening tools, namely the Mini Nutritional Assessment (MNA), the Malnutrition Universal Screening Tool (MUST), and the Subjective Global Assessment (SGA). Depressive symptoms, appetite status, dietary intake and knowledge, and physical activity levels (PAL) were assessed using the Beck Depression Inventory (BDI), the Council on Nutrition Appetite Questionnaire (CNAQ), and the Athens Physical Activity Questionnaire (APAQ), respectively. Multivariate and univariate regression analyses examined associations between malnutrition risk and sociodemographic and clinical variables. K-means clustering was used to identify participant phenotypes based on nutritional and lifestyle characteristics.

**Results:**

The prevalence of malnutrition was low, at 4.5% according to MNA and MUST, but 0% according to the SGA, with poor concordance observed between the three screening tools. Higher depressive symptom scores were independently associated with increased odds of malnutrition, while greater PAL were associated with reduced risk. K-means clustering identified two distinct phenotypic groups; younger participants with lower BMI, and lower PAL showed increased risk of malnutrition according to MUST (*p* = 0.003) and MNA (*p* = 0.006).

**Conclusion:**

Although malnutrition prevalence was low, it was associated with greater depressive symptoms, lower PAL and BMI, and younger age, underscoring the need for comprehensive, multidimensional assessment and integrated care approaches addressing nutritional, psychological, and lifestyle factors.

## Abbreviations

AIDS: acquired immunodeficiency syndrome
APAQ: Athens Physical Activity Questionnaire
ART: antiretroviral therapy
BDI: Beck depression inventory
BMI: body mass index
CI: confidence intervals
CNAQ: Council on Nutrition Appetite Questionnaire
CVD: cardiovascular disease
EAT: Eating Assessment Table
GLIM: Global Leadership Initiative on Malnutrition
HIF-1α: hypoxia-inducible-factor-1-alpha
HIV: human immunodeficiency virus
IFN-γ: interferon-gamma
IQR: interquartile range
MET: metabolic equivalent
MNA: Mini Nutritional Assessment
MUAC: Mid-upper arm circumference
MUST: Malnutrition Universal Screening Tool
NGO: non-governmental organization
OR: odds ratio
PAL: physical activity levels
PWH: people living with HIV
SD: standard deviation
SGA: Subjective Global Assessment
TNF-α: tumor necrosis factor-alpha

## 1 Introduction

Human immunodeficiency virus (HIV) continues to be a major global health challenge, with millions of people affected and ongoing transmission in many regions, despite the availability of effective prevention and treatment strategies [1]. Although substantial progress has been achieved over the past decades, the epidemic remains shaped by persistent inequalities in access to care, delayed diagnosis, and suboptimal treatment engagement, all of which limit the impact of existing interventions [2]. Moreover, as HIV is increasingly managed as a chronic condition, attention has been directed towards non-virological factors that influence long-term health outcomes and quality of life [3]. In this context, undernutrition and wasting remain significant concerns, as they accelerate disease progression while increasing the risk of developing acquired immunodeficiency syndrome (AIDS) [4,5].

Antiretroviral therapy (ART) is a cornerstone in the management of HIV-1 infection [6]. It consists of a combination of three or more antiretroviral agents from at least two different drug classes [6]. The introduction of ART has substantially slowed down disease progression, reduced mortality rates, and induced improvements in the quality of life of people living with HIV (PWH) [[7], [8], [9]]. However, factors associated with disease-related malnutrition and involuntary changes in body weight remain challenging [4,10]. Reduced nutrient intake, malabsorption, HIV-associated weight loss and metabolic disturbances are commonly reported and may be caused by the confluence of infection itself, ART, or hormonal imbalances [[11], [12], [13]]. On the other hand, weight gain and obesity are common and difficult to manage issues following ART initiation [14], reflecting the development of metabolic and immune dysregulation.

In addition to nutritional challenges, PWH are at increased risk of mental health disorders, particularly depression, which has been associated with poor treatment adherence, reduced appetite, and decreased physical activity levels (PAL) [15,16]. These factors may further exacerbate malnutrition and negatively affect overall health outcomes [16]. Although previous studies have examined nutritional status or psychological health independently [4,10,16], the relationship between malnutrition, depressive symptoms, and lifestyle behaviors remains insufficiently explored, particularly in the Greek population of PWH.

Thus, the present pilot study aimed to comprehensively evaluate the prevalence of malnutrition and depressive symptoms, as well as patterns of physical activity and appetite, among PWH on ART, in Greece. Furthermore, it sought to explore potential associations between these factors, providing insights into the complex interplay between nutritional status, mental health, and lifestyle behaviors in this population.

## 2 Methods

### 2.1 Study design and population

This pilot, cross-sectional study recruited PWH through the Non-Governmental Organization (NGO) Kentro Zois, situated in the metropolitan areas of Athens and Thessaloniki, in Greece. Inclusion criteria involved (i) adults (≥18 years old), (ii) tested positive for HIV, (iii) on ART for at least six months, (iv) able to communicate in the Greek language, (v) abstaining from drug use for the past two years, and (vi) willing to participate in the study. A total of 44 individuals provided informed consent and were included in the study. Information on the route of HIV transmission was not recorded, in order to minimize potential discomfort and stigma among participants. The study was initially approved by the Ethics Committee of the Alexander Technological Educational Institute, however, since the entity is no longer existent in the same form, additional approval was granted by the Ethics Committee of the University of Thessaly (approval no. 46/17.06.2026). The characteristics of the study population are presented in Table 1.

**Table 1:** Characteristics of the study population (N = 44).

Variables	*n*, % or mean ± SD, or median (IQR)
**Age** (years)	35.0 (27.8–49.0)^¤^
**Sex** (women/men) (*n*, %)	15 (34.1)/29 (65.9)
**Years since HIV positive diagnosis**	6.0 (3.0–9.0) ^¤^
**BMI** (kg/m^2^)	23.2 (21.2–25.3)^¤^
**BMI tier** (underweight/normoweight/overweight) (*n*, %)	3 (6.8)/28 (63.7)/13 (29.5)
**Waist circumference** (cm)	88.7 ± 12.4^a^
**Central obesity** (*n*, %)	13 (29.5)
**Mid-thigh circumference** (cm)	48.7 ± 5.7^a^
**Chest circumference** (cm)	96.2 ± 9.5^a^
**Calf circumference** (cm)	34.1 ± 4.0^a^
**MUAC** (cm)	22.6 ± 2.4^a^
**Hips circumference** (cm)	100.0 (94.0–105.2) ^¤^
**Neck circumference** (cm)	36.1 ± 3.8^a^
**Triceps skinfold** (mm), (*n* = 15)	17.2 ± 4.3^a^
**Subscapular skinfold** (mm), (*n* = 15)	12.0 (10.0–13.0)^¤^
**Chest skinfold** (mm), (*n* = 29)	6.0 (4.0–11.0)^¤^
**Abdominal skinfold** (mm), (*n* = 29)	14.7 ± 6.7^a^
**Thigh skinfold** (mm), (*n* = 29)	14.5 (11.8–19.3)^¤^
**Cigarettes** (number of cigarettes/day)	0 (0.0–10.0) ^¤^
**HIV-related medication** (ART) (*n*, %)	44 (100)
**Number of children** (0/1/2) (*n*, %)	39 (88.6)/2 (4.5)/3 (6.8)
**Marital status** (single/married/divorced/in a relationship) (*n*, %)	27 (61.4)/5 (11.4)/1 (2.3)/11 (25.0)
**Living status** (alone/with friends/with relatives) (*n*, %)	17 (38.6)/1 (2.3)/26 (59.1)
**Employment** (unemployed/self-employed/employee/retired) (*n*, %)	17 (38.6)/6 (13.6)/15 (34.1)/5 (11.4)
**Education** (primary/secondary/tertiary/postgraduate) (*n*, %)	3 (6.8)/11 (25.0)/23 (52.3)/7 (15.9)
**Comorbidities** (T1DM/T2DM/arterial hypertension/arrhythmia/depression/heart failure/Hodgkin's lymphoma) (*n*, %)	5 (11.4)/5 (11.4)/3 (6.8)/1 (2.3)/3 (6.8)/7 (15.9)/1 (2.3)
**HIV-related comorbidities** (lipodystrophy/Kaposi sarcoma/herpes zoster) (*n*, %)	2 (4.5)/1 (2.3)/6 (13.6)
**Other symptoms** (vomiting/altered taste/fatigue/abdominal pain) (*n*, %)	2 (4.5)/2 (4.5)/1 (2.3)/1 (2.3)

ART: antiretroviral therapy; BMI: body mass index; cm: centimeters; HIV: Human immunodeficiency virus; IQR: interquartile range; kg: kilograms; *n*: number; SD: standard deviation; T1DM: Type 1 diabetes mellitus; T2DM: Type 2 diabetes mellitus.

a mean ± SD; ¤ median (interquartile range).

### 2.2 Anthropometric indices

Anthropometric measurements were obtained by an experienced dietitian (P.P.) following standardized procedures. Body weight and height were measured using a digital floor scale (Kern MPE 200 K-1PEM, Kern, Germany) and a portable stadiometer (Seca 220, Hamburg, Germany), respectively. Body mass index (BMI) was calculated for all patients and was used to categorize participants into distinct weight status tiers, as follows: (i) underweight (BMI <18.5 kg/m^2^), (ii) normoweight (18.5≤ BMI <25 kg/m^2^), (iii) overweight (25 kg/m^2^≤ BMI <30 kg/m^2^).

An unelastic measuring tape was used for measuring waist circumference and determining central obesity (waist circumference of ≥102 cm for men and ≥88 cm for women [17]). In addition, other body circumferences were also assessed at selected anatomical sites. A mid-upper arm circumference (MUAC) of <25.5 cm was used to assess undernutrition based on specific cutoffs for PWH [18]. Body composition was not assessed, in order to limit possible discomfort associated with undressing, as research has indicated poor body image perception among PWH [19] due to lipodystrophy, depression, anxiety, stigma, etc. However, among PWH willing to provide more anthropometric measurements, skinfold thickness was measured using a Slimguide caliper (Creative Health, USA), with measurements taken on the right side of each participant's body.

### 2.3 Malnutrition assessment

Risk for malnutrition was assessed using three distinct tools, namely the Mini Nutritional Assessment (MNA) [20], the Subjective Global Assessment (SGA) [21] and the Malnutrition Universal Screening Tool (MUST) [22].

The MNA is a screening tool [20] used for the evaluation of six domains, including BMI, recent body weight loss, dietary intake, mobility, psychological stress or acute disease, and neuro-psychological issues. The MNA provides a quantitative score that classifies individuals into three categories: normal nutritional status (score: 12–14), at risk of malnutrition (score: 8–11), and malnourished (score: 0–7).

On the other hand, the SGA [21] addresses patient history and clinical examination. The first category includes body weight history, dietary intake, gastrointestinal symptoms, functional capacity, and metabolic stress (i.e., increased nutritional requirements), while the second focuses on the loss of subcutaneous fat, muscle wasting, and the presence of edema or ascites. The classification system for malnutrition employs a three-tiered grading scale. Level A is defined as “well-nourished”, level B as “moderate malnutrition”, and level C as “severe malnutrition”.

Finally, the MUST [22] is based on three core criteria: BMI, unintentional weight loss over the past 3–6 months, and the presence of acute disease, with little or no nutritional intake for more than five days. Each component is scored in a range between 0 and 2, and the sum of the three scores determines the overall risk category. Patients are classified into low (score 0), moderate (score 1), or high risk of malnutrition (score ≥2).

### 2.4 Eating Assessment Table

The Eating Assessment Table (EAT 2008) [23], a structured questionnaire assessing diet quality and nutritional knowledge was also employed. It includes 11 domains: fruit, vegetable, and legumes consumption, food preparation methods and quality of cooking fat, meat intake and meat quality, starchy foods intake and grain quality, dairy consumption and dairy fat content, dietary fat nutrition knowledge and label literacy, alcohol consumption and omega-3 fatty acid intake, dietary habits and eating behaviors, and empty calories consumption. Each domain is scored in a range between 0 and 10. The total score (sum of each component) ranges from 0 to 100, as one of the 11 domains is excluded from the final total. Greater EAT scores indicate better diet quality and greater nutritional knowledge.

### 2.5 Appetite loss

Appetite loss was assessed using the CNAQ (Council on Nutrition Appetite Questionnaire) [24]. The CNAQ is an 8-item questionnaire with answers in a Likert scale scoring between 1 and 5. Total scores range from 8 to 40, with lower scores indicating poorer appetite and a greater need for nutritional intervention. A score <28 may indicate appetite loss and an elevated risk of substantial weight loss.

### 2.6 Depression

Depressive symptoms were evaluated using the Beck Depression Inventory (BDI) [25], which classifies depressive symptoms into six categories: normal (0–9), mild mood disturbance (10–15), borderline clinical depression (16–19), moderate depression (20–29), severe depression (30–39), and extreme depression (40–63). Participants are asked to respond based on the symptoms exhibited during the past two weeks, and each answer is scored on a scale from 0 to 3.

### 2.7 Physical activity

The Athens Physical Activity Questionnaire (APAQ) [26] was used to record PAL of participants. The tool is designed to estimate energy expenditure over the previous week. Participants report the frequency and duration of vigorous, moderate, and walking activities. Each activity is assigned a corresponding metabolic equivalent (MET) value, and overall PAL is calculated as MET-hours per week, by multiplying duration by intensity and summing the PAL of all activities. Based on the total PAL score, individuals are classified into different PAL (low, moderate, or high) categories.

### 2.8 Dietary intake

Patients were asked to provide a 24-h dietary recall through an interview with an experienced dietician (P.P.). The reported dietary intakes were analyzed for macronutrient and micronutrient composition using the Cronometer dietary analysis software (Cronometer Software Inc., Revelstoke, BC, Canada) [27].

### 2.9 Statistical analyses

Continuous variables were assessed for normality using the Shapiro–Wilk test. Depending on their distribution, they are presented as means ± standard deviations (SD) or as medians with the respective interquartile range (IQR). Categorical variables are presented as frequencies and/or percentages. Separate multivariable logistic regression models were constructed due to the limited sample size, with each model examining associations between MNA and different combinations of predictors. Results are reported as odds ratios (OR) with their corresponding 95% confidence intervals (CI). Physical activity energy expenditure (APAQ) was rescaled and analyzed per 100 kcal/day increase to improve the interpretability of the regression coefficients. For outcomes with a limited number of events (MUST), only univariable logistic regression analyses were performed. Due to the pilot nature of the study and the limited sample size, regression analyses were considered exploratory.

Agreement between the three malnutrition screening tools (MNA, MUST, and SGA) was evaluated using percent agreement, Cohen's kappa (for binary classifications), and weighted kappa (for ordinal classifications), with variables dichotomized as follows: MNA (well-nourished *vs*. at risk/malnourished), MUST (low risk *vs*. moderate/high risk), and SGA (well-nourished *vs*. moderate/severe malnutrition). The interpretation of agreement was based on standard kappa thresholds. An alluvial plot was used to visually represent the overlap and transitions between classifications across the three tools, using the {ggalluvial} package.

Exploratory cluster analysis was conducted to identify potential phenotypic patterns. K-means clustering was performed using depressive symptoms (BDI), appetite (CNAQ), PAL (APAQ), diet quality and knowledge (EAT), BMI, and waist circumference. All variables were standardized (z-scores) before clustering. The optimal number of clusters was determined using the elbow method, with k = 2 selected based on interpretability. Cluster validity was further evaluated using the silhouette score. Silhouette analysis was performed using the {cluster} package, and results were visualized using the {factoextra} package, which extends {ggplot2} within a {tidyverse} framework. Continuous variables were assessed for normality within each cluster using the Shapiro–Wilk test. As most variables were not normally distributed, they are presented as medians with interquartile range (IQR), and differences between clusters were assessed using the Wilcoxon rank-sum test. Categorical variables are presented as counts and percentages, and comparisons between clusters were performed using Fisher's exact test or Chi-square test, as appropriate. For malnutrition analyses, variables were dichotomized as previously described. All statistical analyses and graphs were performed in R Studio version 4.5.2 (2026.01.0 + 392), R Foundation for Statistical Computing (Vienna, Austria) [28], with the significance set at *p* < 0.05.

## 3 Results

### 3.1 Prevalence of adiposity, comorbidities, and malnutrition among PWH

The anthropometric results revealed that 6.8% of the participants were classified as underweight, 63.7% had normal body weight, 29.5% were classified as overweight, and none of the participants was classified as having simple obesity. Furthermore, 29.5% of the sample met the criteria for central obesity based on sex-specific waist circumference cutoffs. Based on MUAC classification, 88.6% (*n* = 39) of the participants were classified as undernourished, while 11.4% (*n* = 5) exhibited normal nutritional status. One tenth (1/10) of the participants had diabetes mellitus and 6.8% of the sample suffered from hypertension. A small proportion (6.8%) of PWH reported having a depression diagnosis. With regards to HIV-related symptoms, 13.6% of the participants had a previous herpes zoster infection, 2.3% Kaposi sarcoma and 4.5% of the sample exhibited lipodystrophy.

According to the MNA tool, only two patients (4.5%) were malnourished, and 16 (36.4%) were at risk of malnutrition. The MUST classified two patients (4.5%) as being at high risk of malnutrition and four patients (9.1%) as being at moderate risk. In contrast, the SGA tool did not classify any patients as severely malnourished, while five patients (11.4%) were moderately malnourished. More information is presented in Table 2. Unintentional body weight loss was reported by 9.1% of the sample.

**Table 2:** Prevalence of malnutrition, depression, distinct PAL, and appetite categories among people with HIV on ART (N = 44).

Variables	*n* (%)
**MNA** (well-nourished/at risk of malnutrition/malnutrition)	26 (59.1)/16 (36.4)/2 (4.5)
**MUST** (low risk/moderate risk/high risk of malnutrition)	38 (86.4)/4 (9.1)/2 (4.5)
**SGA** (well-nourished/moderate risk/high risk of malnutrition)	39 (88.6)/5 (11.4)/0 (0)
**BDI** (normal mood fluctuations/mild mood disturbance/borderline clinical depression/moderate depression/severe depression/extreme depression)	26 (59.1)/10 (22.7)/2 (4.5)/4 (9.1)/2 (4.5)/0 (0)
**APAQ** (low/moderate/high PAL)	0 (0)/40 (90.9)/4 (9.1)
**CNAQ** (poor appetite/risk of weight loss/good appetite)	0 (0)/8 (18.2)/36 (81.8)

APAQ: Athens Physical Activity Questionnaire; ART: antiretroviral therapy; BDI: Beck Depression Inventory; CNAQ: The Council on Nutrition Appetite Questionnaire; HIV: human immunodeficiency virus; N: number; MNA: Mini Nutritional Assessment; MUST: Malnutrition Universal Screening Tool; PAL: physical activity level; SGA: Subjective Global Assessment.

**Fig. 1:**
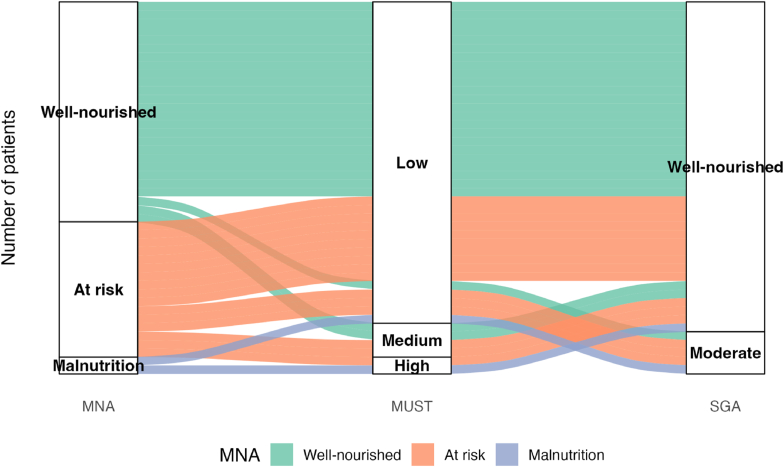
Comparison of nutritional status classification across MNA, MUST, and SGA in people with HIV on ART (N = 44). Colors are based on MNA. Alluvial plot showing the distribution and discordance of patient classification between three commonly used nutritional assessment tools. ART: antiretroviral therapy; HIV: human immunodeficiency virus; MNA: mini nutritional assessment; MUST: Malnutrition Universal Screening Tool; SGA: Subjective Global Assessment. (For interpretation of the references to colour in this figure legend, the reader is referred to the Web version of this article.)

The agreement between the three malnutrition screening tools (MNA, MUST, and SGA) was low. When the tools were used as binary variables (well-nourished and malnourished), the agreement reached 63.6% for both MNA *vs*. MUST and MNA *vs*. SGA, and 86.4% for MUST *vs*. SGA, likely reflecting the predominance of well-nourished participants (Fig. 1). Cohen's kappa indicated weak agreement between MNA *vs*. MUST (κ = 0.162) and MNA *vs*. SGA (κ = 0.207), whereas agreement between the MUST and the SGA was poor (κ = −0.142), suggesting no concordance beyond chance. Weighted kappa for the three-category classification was near zero across all comparisons (MNA *vs*. MUST: κ = 0.003, *p* = 0.813; MNA *vs*. SGA: κ = 0.018, *p* = 0.117; MUST *vs*. SGA: κ = 0.001, *p* = 0.768), indicating poor agreement in grading malnutrition severity (Table 3).

**Table 3:** Concordance between MNA, MUST, and SGA for malnutrition assessment among people with HIV on ART (N = 44).

Comparison	% Agreement	Cohen's Kappa	Weighted Kappa
**MNA *vs*. MUST**	63.6%	0.162 (*p* = 0.167)	0.003 (*p* = 0.813)
**MNA *vs*. SGA**	63.6%	0.207 (*p* = 0.059)	0.018 (*p* = 0.117)
**MUST *vs*. SGA**	86.4%	−0.142 (*p* = 0.345)	0.001 (*p* = 0.768)

The nutritional assessment variables were dichotomized for the logistic regression analyses as follows: MNA (well-nourished *vs*. at risk of malnutrition/malnourished), MUST (low risk *vs*. moderate/high risk), and SGA (well-nourished *vs.* moderately/severely malnourished). ART: antiretroviral therapy; HIV: human immunodeficiency virus; MNA: mini nutritional assessment; N: number; MUST: Malnutrition Universal Screening Tool; SGA: Subjective Global Assessment.

### 3.2 Depression symptomatology

According to the BDI, ten PWH (22.7%) experienced mild mood disturbance, two (4.5%) exhibited borderline clinical depression, four (9.1%) suffered from moderate depression, and two (4.5%) had severe depression (Table 2). Asides from the BDI, depression diagnosis was reported by 6.8% of the sample.

### 3.3 Appetite, diet quality, and nutritional knowledge

Concerning appetite, 36 patients (81.8%) exhibited good appetite based on the CNAQ, whereas the remaining eight (18.2%) were identified as being at risk of weight loss.

The total EAT score of participants was 45.4 ± 12.7. Scores varied across domains, with greater values observed for meat intake and meat quality, food preparation methods and cooking fat quality, and starchy foods and grain quality, while lower scores were observed for dairy consumption, dairy fat content and empty calories intake (Table 4). The intake-related subscale (food groups and cooking methods) score of the sample was estimated at 20.4 ± 5.65, whereas the dietary knowledge and behavior-related subscale (nutritional knowledge and specific habits) had a score of 25.0 ± 8.64, both indicating suboptimal dietary intake and nutritional knowledge.

**Table 4:** Scoring of each Eating Assessment Table (EAT 2008) question (N = 44).

Q	Category	Score
1	Fruit consumption	4 (2–6)^¤^
2	Vegetables consumption	4 (2–6)^¤^
3	Legumes consumption	4 (2–4)^¤^
4	Food preparation methods and cooking fat quality	8 (8–10)^¤^
5	Meat intake and meat quality	7 (5–9)^¤^
6	Starchy foods and grain quality	6 (4.75–8)^¤^
7	Dairy consumption and dairy fat content	2 (0–4)^¤^
8	Dietary fat nutrition knowledge and label literacy	6 (4–8)^¤^
9	Alcohol consumption and omega-3 fatty acid intake	2.25 (1–3.63)^¤^
10	Dietary habits and eating behaviors	6 (3.5–8)^¤^
11	Empty calories consumption	−3 (–6–0)^¤^

1–4	Food groups intake and cooking methods	20.4 ± 5.65†
5–11	Nutrition knowledge and specific habits	25.0 ± 8.64†

	Total EAT score	45.4 ± 12.7†

EAT: Eating Assessment Table; Q: Question. † mean ± SD; ^¤^ median (interquartile range).

According to the previous 24-h dietary recall, daily energy intake was 1664.5 ± 625.9 kcal, with a protein intake of 67.8 ± 28.2 g/day. Carbohydrate and total fat intake were 184.0 ± 71.6 g/day and 72.0 ± 37.9 g/day, respectively. Dietary fiber intake was low (15.5 ± 8.2 g/day). Micronutrient intake showed suboptimal levels for several nutrients, including vitamin D (1.6 ± 2.2 μg/day), vitamin E (6.2 ± 3.7 mg/day), and folate (235.3 (IQR 167.7) μg/day). Calcium intake was 750.2 (IQR 392.1) mg/day, while Iron intake was 9.9 ± 3.9 mg/day. Intake of fruit, vegetables, and fish was low, at 105.0 (IQR 220.3) g/day, 132.5 (IQR 230.0) g/day, and 0 g/day, respectively. Alcohol consumption was negligible. More details can be found in Supplementary Table 1.

### 3.4 Physical activity

Regarding physical activity, most patients (*n* = 40, 90.9%) demonstrated moderate PAL, while the remaining (9.1%) were classified as having high PAL.

### 3.5 Regression analyses

Univariate logistic regression analyses were performed to examine crude associations between independent variables and nutritional status outcomes (data not shown). Variables showing relevant associations and/or biological plausibility were subsequently included in multivariable logistic regression models to adjust for potential confounders. Multivariable logistic regression analyses (Table 5) showed that greater depression scores were independently associated with increased malnutrition odds according to the MNA tool when adjusted for appetite (Model 1: OR: 1.14, 95% CI: 1.04 to 1.30, *p* = 0.017) and PAL (Model 2: APAQ (per 100 kcal/day increase in daily energy expenditure) OR: 0.73, 95% CI: 0.55 to 0.88, *p* = 0.007). Greater PAL was additionally associated with reduced odds for malnutrition, when accounting for appetite (OR: 0.81, 95% CI: 0.66 to 0.94, *p* = 0.013). In a fourth model, greater PAL (OR: 0.78, 95% CI: 0.64 to 0.91, *p* = 0.005) and improved diet quality (OR: 0.93, 95% CI: 0.85 to 0.99, *p* = 0.032) were independently associated with reduced malnutrition risk.

**Table 5:** Univariable and multivariable logistic regression models for malnutrition of people with HIV on ART (N = 44).

	Outcome	Predictor	OR (95% CI)	*p* value
**Multivariable**	**MNA (Model 1)**	CNAQ	0.84 (0.64–1.08)	0.19
		BDI	1.14 (1.04–1.30)	0.017∗
	**MNA (Model 2)**	BDI	1.24 (1.09–1.48)	0.006∗
		APAQ (per 100 kcal/day increase)	0.73 (0.55–0.88)	0.007∗
	**MNA (Model 3)**	CNAQ	0.79 (0.60–1.00)	0.07
		APAQ (per 100 kcal/day increase)	0.81 (0.66–0.94)	0.013∗
	**MNA (Model 4)**	APAQ (per 100 kcal/day increase)	0.78 (0.64–0.91)	0.005∗
		EAT	0.93 (0.85–0.99)	0.032∗
**Univariate**	**MUST**	APAQ (per 100 kcal/day increase)	0.57 (0.33–0.81)	0.010∗
		CNAQ	0.82 (0.60–1.10)	0.20
		Years since HIV diagnosis	0.90 (0.70–1.04)	0.30
		EAT	1.00 (0.93–1.07)	0.925

APAQ: Athens Physical Activity Questionnaire; ART: antiretroviral therapy; BDI: Beck depression inventory; CI: confidence intervals; CNAQ: The Council on Nutrition Appetite Questionnaire; EAT: Eating Assessment Table; HIV: Ηuman immunodeficiency virus; MNA: Mini Nutritional Assessment; MUST: Malnutrition Universal Screening Tool; N: number; OR: odds ratio. ∗*p* < 0.05.

In univariable analyses for the MUST, greater PAL (APAQ, per 100 kcal/day increase in daily energy expenditure) was similarly associated with reduced malnutrition odds (OR: 0.57, 95% CI: 0.33 to 0.81, *p* = 0.010). No associations were observed for appetite, or years since HIV positive diagnosis. Given the pilot nature of the study and the limited number of malnutrition observations, regression findings should be interpreted as exploratory.

### 3.6 Clusters

**Fig. 2:**
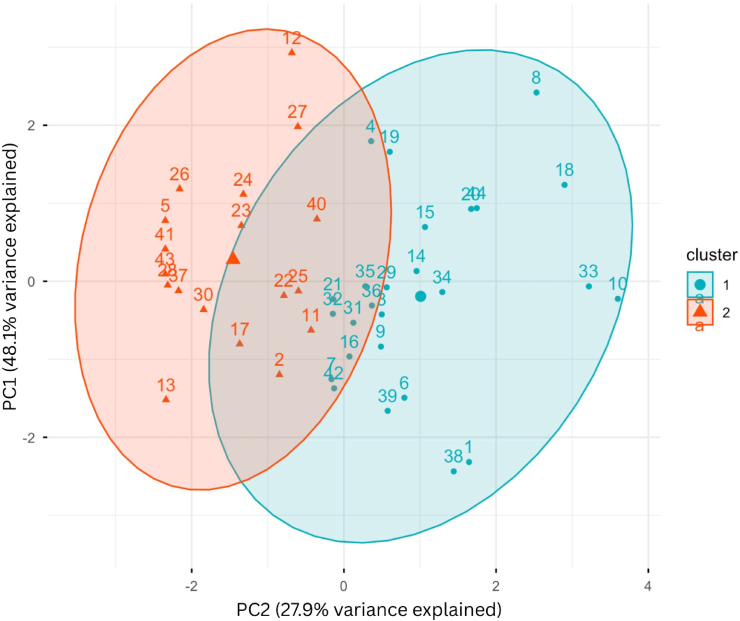
K-means clustering of people living with HIV on ART based on nutritional, psychological, and anthropometric variables (N = 44). ART: antiretroviral therapy; HIV: human immunodeficiency virus; PC: principal component analysis.

K-means cluster analysis identified two distinct groups among participants. Cluster 1 (*n* = 26) was characterized by greater PAL, older age, higher BMI, better diet quality and larger waist circumference compared to Cluster 2 (*n* = 18) (Table 6). No differences were observed between clusters regarding depressive symptomatology or appetite scores. Silhouette analysis indicated modest cluster separation (mean silhouette width = 0.22), suggesting limited differentiation between groups (Fig. 2). A sensitivity analysis excluding the EAT score resulted in a slight increase in mean silhouette width (from 0.22 to 0.26), while cluster composition and interpretation remained unchanged.

**Table 6:** Characteristics of clusters of people with HIV on ART, identified by k-means analysis (N = 44).

Variable	Cluster 1 (*n* = 26)	Cluster 2 (*n* = 18)	*p* value
Depression (BDI score)	9 (5–15.5)	7 (4.5–12.75)	0.658
Appetite (CNAQ score)	32 (30.25–33)	31 (29–32)	0.224
EAT	44.5 (37.5–53)	44 (37.5–53)	0.89
Physical activity (APAQ score)	2471 (2281–2856.5)	1786 (1546–2038)	<0.001∗
BMI (kg/m^2^)	24.7 (23.49–26.73)	20.44 (19.81–22.34)	<0.001∗
Waist circumference (cm)	92.5 (90–102.5)	77 (73–83.5)	<0.001∗
Age (years)	44.5 (37.5–53.25)	29.5 (25.25–34.75)	0.002∗
Years since HIV diagnosis	7.5 (3.25–15.5)	6 (3–7.75)	0.122
MNA (at risk or malnourished)	6/26 (23.1%)	12/18 (66.7%)	0.006∗
MUST (moderate or high risk)	0/26 (0%)	6/18 (33.3%)	0.003∗
SGA (moderate or severe)	3/26 (11.5%)	2/18 (11.1%)	1.000

APAQ: Athens Physical Activity Questionnaire; ART: antiretroviral therapy; BDI: Beck Depression Inventory; BMI: body mass index; cm: centimeters; CNAQ: The Council on Nutrition Appetite Questionnaire; EAT: Eating Assessment Table; HIV: human immunodeficiency virus; MNA: Mini Nutritional Assessment; MUST: Malnutrition Universal Screening Tool; N: number; SD: standard deviation; SGA: Subjective Global Assessment. All continuous variables are presented as medians (interquartile range). *p* values are derived from Wilcoxon rank-sum test for non-normally distributed data; *t*-test if normally distributed. Categorical variables: *n* (%), *p* values from Fisher's exact tests. ∗*p* < 0.05.

Cluster membership was also related to nutritional risk. According to the MUST classification, all PWH at moderate/high malnutrition risk were fitted in Cluster 2 (33.3%), whereas no individual in Cluster 1 was classified as being at risk for developing malnutrition (*p* = 0.003). Similarly, malnutrition classification based on the MNA revealed a greater prevalence of malnutrition risk in Cluster 2 compared to Cluster 1 (66.7% vs. 23.1%, *p* = 0.010). No association was observed between cluster membership and SGA classification (*p* = 1.00). Age also differed between clusters (*p* = 0.002), with individuals in Cluster 1 being older, while no difference was observed for years since HIV positive diagnosis (*p* = 0.12).

## 4 Discussion

The present pilot study examined the prevalence and determinants of malnutrition in PWH on ART and identified distinct behavioral and anthropometric phenotypes. The study identified a moderate prevalence of overweight, reaching 29.5% of the sample. On the other hand, the prevalence of malnutrition was low among participants; however, MUAC-based assessment showed that undernutrition was predominant among PWH. Greater depressive symptoms were associated with increased malnutrition odds, whereas higher PAL consistently reduced malnutrition risk, independently of appetite sensation or years since HIV diagnosis. Two distinct participant phenotypes were identified, a “higher BMI–active” cluster and a “lean–low PAL” cluster.

Weight gain and obesity are common issues among patients on antiretroviral regimens and have proven to be issues difficult to manage [14]. HIV infection and ART exert diverse effects on adipocytes, altering adipose tissue size, quantity, distribution, and energy storage, while leading to lipodystrophy [29]. Increased adiposity has been suggested to occur on antiretroviral therapies due to increased adipocyte size, elevated oxidative stress and mitochondrial dysfunction [30]. In parallel, specific ART regimens have been suggested to induce a more profound weight gain (dolutegravir) [31], associated with a hypertrophic, insulin-resistant state, due to the confluence of increased hypoxia-inducible-factor-1-alpha (*HIF-1α*) gene expression and the inhibition of brown fat adipogenesis [32,33]. Furthermore, antiretroviral strategies have been shown to inhibit adipocyte beiging as a result of blocked estrogen receptor signaling, thereby increasing adipocyte accumulation and weight gain [14]. Among PWH with advanced disease, weight gain reflects immune reconstitution, and is associated with reduced mortality [34]. On the other hand, weight gain in PWH who were previously obese is associated with a greater risk for cardiovascular disease (CVD) and diabetes [35]. Interestingly, those exhibiting lower CD4 baseline levels and higher viral load tend to experience greater body weight increases, indicating underlying metabolic dysregulation [34]. To combat weight gain, behavioral lifestyle treatment (diet and exercise), switching ART therapies and the use of new anti-obesity medication has been proposed [36,37].

Irrespectively of weight gain, wasting remains an issue of concern in HIV. Poor nutritional status multiplies the risk for developing opportunistic infections, due to immunosuppression [38], while unintentional weight loss is particularly common in a subgroup of PWH, those with controlled viremia [39]. Herein, 9.1% of participants reported HIV-associated weight loss. Unintended weight loss is often associated with loss of muscle mass, wasting, frailty, sarcopenia, reduced physical functioning, and cognitive decline [13]. According to research, PWH receiving ART for less than a year have a 2.68-fold greater risk of developing undernutrition compared to individuals receiving it for more than a year [4]. Accordingly, younger individuals and those in the early stages of ART appear to be particularly vulnerable to developing malnutrition [4]. In the present study, although included participants received ART for at least six months, treatment duration was not further stratified, limiting the ability to assess any possible associations between ART and nutritional status. While ART and, in particular, use of second-generation integrase strand transfer inhibitors is generally associated with improvements in nutritional status [40,41] and weight gain, it may also increase nutrient requirements and metabolic stress [[42], [43], [44]]. In parallel, the OPERA cohort [45] has revealed that not all PWH who receive ART experience weight gain. Some individuals on ART without underlying comorbidities may still suffer from HIV-weight loss as the residue of medication side effects, nutritional deficiencies, or other factors [12]. Subsequently, early nutritional recovery with ART may be modest, and some PWH may remain undernourished despite treatment [46], with persistent micronutrient deficiencies [[42], [43], [44]]. To combat malnutrition and muscle wasting, the evidence indicates that protein supplementation is effective in improving muscle mass, CD4 count and body weight in PWH [47,48].

Herein, MNA-identified malnutrition risk was mainly attributable to psychological stress or acute disease in the past three months, mild cognitive impairment, and low BMI. On the other hand, malnutrition screening with MUST primarily reflected changes in BMI, whereas the SGA captured disease-related stress alongside muscle or fat loss (data not shown). Interestingly, despite a relatively high percentage agreement between tools, Cohen's kappa was low. This apparent paradox likely reflects the predominance of well-nourished participants in the sample, which reduces variability and limits the ability of agreement statistics to capture true concordance beyond chance. Clinically, this highlights the limitations of relying on a single tool for nutritional assessment in PWH and suggests that multidimensional or combined approaches may be necessary to identify at-risk individuals accurately, particularly when malnutrition arises from domains not captured by purely anthropometric or clinical measures. Common screening tools might not have the sensitivity to detect issues associated with changes in visceral fat accumulation, protein loss, lipodystrophy and MUAC-based undernutrition, observed in HIV [49]. For this, the modified SGA-HIV questionnaire, combining anthropometry with lab results, has been suggested by some researchers for determining nutritional risk in HIV [50]. However, it does not account for the parameters associated with HIV-weight loss identified in the recent relevant consensus report [13]. In PWH, metabolic abnormalities such as lipodystrophy can mask underlying muscle wasting [51], thus, standardized or triangulated assessment frameworks incorporating functional measures, objective anthropometrics, or the Global Leadership Initiative on Malnutrition (GLIM) criteria that also combine functional ability [52], may improve early identification and guide tailored interventions to maintain nutritional health.

In addition, patients receiving ART have been shown to exhibit lower depression rates, in comparison to PWH on other therapeutic regimens (26.7% vs. 37.3%) [53]. Herein, 40.8% of participants presented some degree of mood disturbance, with 18.1% exceeding the threshold for clinical depression. Depression was identified as an independent predictor of malnutrition risk, in line with previous studies demonstrating an association between depression and undernutrition among PWH on ART (BMI <18.5 kg/m^2^), in comparison to those having a normal BMI [16]. Mechanistically, depression leads to poorer appetite, which can further propel insufficient nutrient intake and malnutrition [16,54]. However, the present study failed to observe associations between depression and undernutrition. It is noteworthy however, that most studies reporting a strong relationship between depression traits and undernutrition originate from African settings [4,10,16], where socioeconomic constraints and food insecurity are more prevalent than in Greece [55]. The lack of observed differences in the BDI and CNAQ scores between clusters in the present study further suggests that behavioral and anthropometric factors may play a more prominent role in defining malnutrition risk in PWH than psychological or appetite-related parameters alone.

Appetite loss consists of a common symptom among PWH, linked to HIV wasting syndrome. Herein, appetite of participants was generally good, consistent with previous reports showing that PWH often experience improved appetite following ART initiation [56]. In PWH, chronic immune activation and elevated pro-inflammatory cytokines such as tumor necrosis factor-alpha (TNF-α) and interferon-gamma (IFN-γ) [57], may contribute to appetite disturbances, further leading to involuntary weight loss, nutrient deficiencies, and reduced energy levels [42]. Proper appetite regulation is also associated with lower mortality risk, highlighting its importance in maintaining nutritional status and overall health in this population [56].

Herein, most patients exhibited moderate PAL. However, PAL was independently associated with malnutrition risk. Cluster analysis further demonstrated that individuals at greater risk of malnutrition were characterized by lower PAL, lower BMI, and a younger age. While existing literature presents a bifurcated view associating physical inactivity with both lower BMI in PWH [58] and obesity in a mixed population of PWH and AIDS [59], our findings align with the former. These findings suggest that lower BMI and reduced PAL may represent key indicators of nutritional vulnerability in PWH, independent of depressive symptoms or changes in appetite and hunger.

The clinical significance of a low BMI in the context of HIV is often rooted in reduced musculoskeletal integrity. Previous research has consistently associated lower BMI with diminished grip strength [60], a proxy for the depletion of lean body mass common in AIDS [61]. This pathology is central to HIV wasting syndrome, a multi-factorial condition observed in advanced immunosuppression in untreated or late-stage HIV infection (AIDS) driven by inadequate caloric intake, malabsorption, and altered nutrient utilization [43]. Furthermore, the metabolic demands of chronic immune activation and comorbid conditions can induce a state of hypermetabolism, significantly increasing resting energy expenditure and accelerating the loss of both adipose and lean tissue [43]. Addressing this functional decline is critical for long-term clinical outcomes. Evidence suggests that structured exercise interventions, specifically aerobic or combined resistance training performed at least thrice weekly for a minimum of five weeks, is not only safe but highly effective in enhancing cardiorespiratory fitness, muscular strength, and overall body composition [62,63]. Furthermore, while obesity-related functional decline consists of a concern in the ART era, our findings highlight the fact that the low-BMI wasting phenotype remains a potent driver of reduced PAL. This underscores the necessity of a nuanced clinical approach that addresses both extremes of the body composition spectrum to optimize functional outcomes in PWH.

Regarding age, Tegene *et al*. showed that physically inactive patients were of younger age [64], while a meta-analysis revealed that the risk of malnutrition is greater among younger PWH. This age-related disparity may be attributable to a lower degree of 'treatment literacy' and reduced engagement with comprehensive HIV care pathways during the early stages post-diagnosis [4]. In contrast, older individuals may demonstrate greater acceptance and adherence to healthcare recommendations over time [4]. Nevertheless, it must be noted that the present study did not collect data regarding the duration of ART; thus, the observed association of PAL with younger age warrants further investigation.

PWH are at a greater risk of developing CVD due to underlying pathophysiological mechanisms, including chronic inflammation [65], as well as the side effects of ART, which may contribute to cardiac dysfunction through mitochondrial toxicity and metabolic disturbances, comprising lipid accumulation and insulin resistance [66,67]. In this context, physically inactive individuals with low BMI may further exacerbate metabolic stress, while reducing physiological resilience. Low BMI does not necessarily confer cardiovascular protection, as micronutrient inadequacies and reduced muscle reserves, alongside low PAL, may blunt cardiometabolic resilience, contributing to elevated CVD risk despite the absence of overt adiposity [[42], [43], [44]].

While the Academy of Nutrition & Dietetics has underlined the importance of nutrition as an important component of HIV care, recommending that PWH receive nutritional counseling and assurance of food and nutrition security [68], challenges remain, particularly in low-income settings, where limited resources and infrastructure can impede effective implementation [42,48]. The current study also revealed that PWH exhibit suboptimal diet quality and nutritional knowledge, underscoring the importance of targeted nutritional education and dietary interventions to improve dietary intake. Intake of key micronutrients, including vitamin D, vitamin C, folate, vitamin E, Calcium, Magnesium, Potassium, and Zinc, was substantially below recommended levels among present participants, while intake of dietary fiber was also markedly insufficient. In contrast, saturated fat intake exceeded the recommended limits. These findings are in line with previous research reporting suboptimal intake of vitamin D, folate, fiber, as well as fruit and vegetables among PWH, alongside an increased consumption of saturated fat and Sodium [[69], [70], [71]]. Approximately one in every three patients with HIV has been reported to follow a diet of poor quality, which may adversely affect body weight and body composition [71].

Nutritional interventions with oral nutrient supplements including Selenium, Zinc, Copper, Iron, and vitamins A and D, have an impact on oxidative stress, immunological response, and metabolic homeostasis, highlighting their potential beneficial role as adjuvant therapies [42,72]. In addition, protein-energy-fortified macronutrient supplements led to changes in nutritional status and immunological response in PWH [47]. Nevertheless, two systematic reviews indicated that nutritional intervention programs led to modest improvements in weight-related outcomes but were limited by high default and non-response rates, as well as resource constraints, particularly regarding food security [73,74]. Despite these limitations, nutritional support provided alongside ART may still confer clinical benefits, including promoting weight gain, enhancing immune recovery, and improving PAL, particularly among patients who present with weight loss at ART initiation [72]. According to Keshani [75] a strong association exists between CD4 count and the Western dietary pattern, with each unit increase in the Western diet score increasing the odds of CD4 < 500 by 57%. Nutrition and immunity are intertwined [76] since the gut consists of the largest immune organ, housing 70–80% of all immune cells [77]. In this manner, consuming a healthy and balanced diet may confer improvements in immune response.

### 4.1 Limitations of the study

The present study has several limitations. First, its cross-sectional design precludes causal inferences. Second, the relatively small sample size and the pilot nature of the study limit statistical power and require cautious interpretation of the findings. Thus, the results should be considered exploratory and hypothesis-generating and should be confirmed in studies using larger samples. Also, the limited sample size restricted the number of variables included in the multivariate regression analyses, which may have resulted in residual confounding. Additionally, PAL was assessed using self-reported measures, which are subject to recall and social desirability biases and may be less accurate than objective methods, such as motion sensors or pedometers. Furthermore, information regarding ART regimens, CD4 cell count, viral load and food insecurity was not systematically collected. Moreover, dietary intake was assessed using a single previous 24-h dietary recall, which may not reflect usual dietary intake and is subject to recall bias and day-to-day variability. Although the cluster structure showed only moderate separation, the consistency of findings across multiple nutritional assessment tools supports the potential clinical relevance of these phenotypes. Nevertheless, larger studies are required to confirm these results and to further explore their implications for targeted nutritional and behavioral interventions.

## 5 Conclusion

The prevalence of malnutrition among PWH in the present pilot sample was low. However, the findings indicate that appropriate nutritional support is necessary for PWH, considering their underlying nutritional status, suboptimal nutritional knowledge, dietary intake of moderate quality, comorbidities, and disease-related nutritional recommendations [38]. Additionally, a comprehensive assessment of nutritional status based on the ABCDE framework is required for all PWH. Given the multifactorial nature of malnutrition in PWH, as well as its adverse clinical outcomes and potential to accelerate disease progression if left untreated, an integrated approach that considers nutritional status, mental health, and lifestyle factors is crucial for effective management and care.

## Ethical considerations

The protocol of the study was initially approved by the Ethics Committee of the Alexander Technological Educational Institute, which has since changed identity under the name International Hellenic University. The study protocol was subsequently re-approved by the Ethics Committee of the University of Thessaly (approval no. 46/17.06.2026). The study was conducted in accordance with the principles of the Declaration of Helsinki, and all participants provided informed consent prior to participation.

## Consent for publication

Informed consent was obtained from all participants.

## Data availability

The datasets collected for this manuscript are accessible from the corresponding author upon reasonable request.

## Funding

This research did not receive any specific grant from funding agencies in the public, commercial, or not-for-profit sectors.

## CRediT authorship contribution statement

**Panagiotis Provias:** Data curation, Investigation. **Eleni C. Pardali:** Formal analysis, Visualization, Writing – original draft, Writing – review & editing. **Aleks Pepa:** Formal analysis. **Arriana Gkouvi:** Writing – review & editing. **Dimitrios Poulimeneas:** Writing – review & editing. **Christina Tsigalou:** Resources, Writing – review & editing. **Maria Dalamaga:** Resources, Writing – review & editing. **Dimitrios G. Goulis:** Methodology, Writing – review & editing. **Dimitrios P. Bogdanos:** Methodology, Writing – review & editing. **Maria G. Grammatikopoulou:** Methodology, Project administration, Resources, Supervision, Writing – original draft, Writing – review & editing.

## Declaration of conflicting interest

Given her role as co-Editor-in-chief, Prof Maria Dalamaga had no involvement in the peer review of this article and had no access to information regarding its peer review. Full responsibility for the editorial process regarding this article was delegated to another journal editor. The rest of the authors declare that they have no known competing financial interests or personal relationships that could have appeared to influence the work reported in this paper.
