# Supplementary material for: Prevalence and associated factors of malnutrition among adults living with HIV on ART: a pilot study
Source: Metabol Open. 2026 Jun 30;31:100483. doi: 10.1016/j.metop.2026.100483 (PMC13355231; doi:10.1016/j.metop.2026.100483)
Supplement: Multimedia component 1 [file mmc1.docx]

**Supplementary Table 1**. Daily dietary intake of PWH (N=44).

| Nutrient | Mean ± SD or Median (IQR) |
| --- | --- |
| Energy (kcal/d) | 1664.5 ± 625.9† |
| Protein nntake (g/d) | 67.8 ± 28.2† |
| Carbohydrate intake (g/d) | 184.0 ± 71.6† |
| Monosaccharides intake (g/d) | 10.3 (30.5)^¤^ |
| Disaccharides intake (g/d) | 20.8 (23.4)^¤^ |
| Dietary fiber (g/d) | 15.5 ± 8.2† |
| Total fat intake (g/d) | 72.0 ± 37.9† |
| Saturated fatty acids (g/d) | 24.1 ± 14.7† |
| MUFA (g/d) | 23.7 (13.5)^¤^ |
| PUFA (g/d) | 6.4 (8.3)^¤^ |
| Trans fatty acids (g/d) | 0.5 (1.9)^¤^ |
| Cholesterol (mg/d) | 190.6 (188.1)^¤^ |
| Vitamin A (RE/d) | 391.3 (360.0)^¤^ |
| Thiamine (Vitamin B1) (mg/d) | 1.1 (0.5)^¤^ |
| Riboflavin (Vitamin B2) (mg/d) | 1.5 ± 0.6† |
| Niacin (Vitamin B3) (mg/d) | 15.1 (14.9)^¤^ |
| Vitamin B6 (mg/d) | 1.1 (0.8)^¤^ |
| Vitamin B12 (μg/d) | 3.1 (3.0) ^¤^ |
| Vitamin C (mg/d) | 49.5 (113.2)^¤^ |
| Vitamin D (μg/d) | 1.6 (2.2)^¤^ |
| Vitamin E (mg/d) | 6.2 ± 3.7† |
| Folate (Vitamin B9) (μg/d) | 235.3 (167.7)^¤^ |
| Pantothenic acid (Vitamin B5) (mg/d) | 2.9 ± 1.5† |
| Vitamin K (μg/d) | 19.3 (34.3)^¤^ |
| Calcium (mg/d) | 750.2 (392.1)^¤^ |
| Copper (mg/d) | 0.7 (0.6)^¤^ |
| Iron (mg/d) | 9.9 ± 3.9† |
| Magnesium (mg/d) | 186.5 ± 82.4† |
| Phosphorus (mg/d) | 946.7 ± 400.6† |
| Potassium (mg/d) | 1989.2 ± 848.3† |
| Selenium (μg/d) | 63.8 (57.9)^¤^ |
| Sodium (mg/d) | 1958.1 (1631.0)^¤^ |
| Zinc (mg/d) | 7.5 ± 3.8† |
| Omega-3 fatty acids (g/d) | 0.6 (0.5)^¤^ |
| Omega-6 fatty acids (g/d) | 5.00 (6.8)^¤^ |
| Alcohol intake (g/d) | 0.0 (0.0)^¤^ |
| Total fruit intake (g/day) | 105.0 (220.3)^¤^ |
| Total vegetable intake (g/day) | 132.5 (230.0)^¤^ |
| Total meat intake (g/day) | 110.0 (132.5)^¤^ |
| Total fish intake (g/day) | 0.0 (0.0)^¤^ |
| Total dairy intake (g/day) | 103.5 (241.5)^¤^ |

IQR: interquartile range; PWH: people living with HIV; SD: standard deviation. † mean ± SD; ^¤^ median (IQR).
